# Efficacy of Photobiomodulation Therapy Utilizing 808 nm and 660 nm Alone and in Combination for Treatment of Paresthesia in Rats

**DOI:** 10.3390/biomedicines13010065

**Published:** 2024-12-30

**Authors:** Ehsan Hajesmaelzade, Mohammad Mohammadi, Sina Kakooei, Luca Solimei, Stefano Benedicenti, Nasim Chiniforush

**Affiliations:** 1 Department of Periodontics, Kerman Dental School, Kerman University of Medical Sciences, Kerman 761691355, Iran; ehsan.hajesmaelzade@gmail.com; 2 Oral and Dental Diseases Research Center, Kerman Dental School, Kerman University of Medical Sciences, Kerman 761691355, Iran; 3 Endodontology Research Center, Kerman University of Medical Sciences, Kerman 761691355, Iran; s.kakooei@yahoo.com; 4 Department of Surgical Sciences and Integrated Diagnostics, University of Genoa, Viale Benedetto XV, 6, 16132 Genoa, Italy; lucasolimei@hotmail.it (L.S.); benedicenti@unige.it (S.B.); nasimch2002@yahoo.com (N.C.); 5 Dentofacial Deformities Research Center, Research Institute for Dental Sciences, Shahid Beheshti University of Medical Sciences, Tehran 19839-63113, Iran

**Keywords:** photobiomodulation therapy, paresthesia, rats, nerve injury

## Abstract

**Background/Objectives**: This study assessed the efficacy of photobiomodulation therapy (PBM) by 808 nm and 660 nm alone and in combination for the treatment of paresthesia in rats. **Methods**: This animal study was conducted on 36 adult male Wistar rats. After general anesthesia, the facial nerve of the right side of the face of rats was surgically exposed and pinched, returned in place, and sutured. The rats were randomly assigned to six groups (*n* = 6) of (I) no-intervention (control), (II) no-laser, (III) 808 nm laser (250 mW, 4 W/cm^2^, 20 s, 8 J/cm^2^, (IV) 660 nm laser (150 mW, 0.25 W/cm^2^, 32 s, 8 J/cm^2^, (V) 808 nm plus 660 nm laser with the original settings, and (VI) 808 nm plus 660 nm laser with half of the time and energy density. After 16 days, a biopsy sample was taken from the nerve injury site and underwent histological, histometric, and immunohistochemical assessments. **Results**: Significantly lower edema and congestion were seen in the combined laser group with original settings (*p* < 0.05); this group had no significant difference with the control group regarding degenerative changes of the nerve fibers and Schwann cells (*p* > 0.05). The 660 nm, and combined laser groups, had a significantly lower accumulation of inflammatory cells (*p* < 0.05). The number of blood vessels in combined laser groups was significantly lower than that in the no-laser group (*p* < 0.05). **Conclusions**: The results showed the positive efficacy of PBM by 808 nm and 660 nm lasers in resolution of inflammation and reduction of degenerative changes of Schwann cells and nerve fibers.

## 1. Introduction

Nerve injury may occur in several dental procedures such as anesthetic injection, root canal therapy, tooth extraction, dentoalveolar surgery, implant surgery, orthognathic surgery, tumor resection, temporomandibular joint surgery, or trauma, causing paresthesia, which may be transient or permanent [1,2,3,4]. Paresthesia leads to a reduction or total loss of the muscle function, leading to muscle atrophy and some degrees of disability, and can adversely affect the quality of life [5,6].

Several approaches have been proposed for management of neurosensory disturbances such as systemic pharmaceutical therapy, physiotherapy, local electric stimulation, acupuncture, reparative nerve surgery, and low-level laser therapy (LLLT), also known as photobiomodulation therapy (PBMT) [7,8,9]. PBMT has gained increasing popularity due to its non-invasiveness, unique properties, and optimal efficacy. PBMT for management of nerve injury and paresthesia was first proposed in 1978, and has become increasingly popular since then [10,11,12,13].

Several parameters such as the laser wavelength, type of the affected nerve, and irradiation parameters can affect the therapeutic efficacy of PBMT. According to the literature, 361 nm to 1064 nm wavelengths of laser are often used for this purpose with promising results [14]. Ayranci et al. [10] showed the optimal efficacy of a Nd:YAG laser with 1064 nm wavelength and 8 J/cm^2^ energy density for the reduction of neurosensory disturbances following inferior alveolar nerve injury during implant surgery. Esmaeelinejad et al. [15] reported significant improvement in the mechanoreceptor response 12 months after treatment with a 810 nm low-level laser with 8.4 J/cm^2^ energy density. Another study showed the significant neurosensory improvement of the compressed inferior alveolar nerve in rats through the irradiation of 810 and 980 nm diode lasers; the 810 nm laser led to a greater improvement in the modulation of immunological biomarkers [16]. Nonetheless, no consensus has been reached on a specific laser irradiation protocol for the maximum enhancement of the recovery from paresthesia. To the best of the authors’ knowledge, no previous study has addressed the effect of PBMT by the combined use of different laser wavelengths on edema and congestion. Thus, this study aimed to assess the efficacy of PBMT by 808 nm and 660 nm lasers alone and in combination for the treatment of paresthesia in rats.

## 2. Materials and Methods

This animal study was conducted on 36 adult male Wistar rats weighing 250 to 300 g, with an approximate age of 3 months. The study was conducted in accordance with the guidelines for the care and use of laboratory animals, and the study protocol was approved by the ethics committee of Kerman University of Medical Sciences IR.kMU.AEC.1402.053.

### 2.1. Sample Size

The sample size was calculated to be 36 rats according to previous studies [6,17].

### 2.2. Surgical Procedure

The rats were allowed 2 weeks for the purpose of acclimation. They were kept in standard cages and had ad libitum access to food and water, with 12-h light/12-h dark cycles. The temperature was adjusted to 25–30 °C.

Prior to surgery, the surgical site was shaved and disinfected with povidone-iodine. The rats were generally anesthetized with 90 mg/kg ketamine (Alfasan, Woerden, Holland) and 90 mg/kg xylazine (Alfasan, Woerden, Holland). Under sterile conditions, a 1-cm incision was made in the skin of the right side of the face along the line connecting the tragus to the lip corner using a #15 surgical scalpel. After exposure of the facial nerve, it was held still with a hemostat and another hemostat with a smooth tip was used to pinch the mid-point of the nerve for 30 s with constant pressure. The injured nerve was then placed back in its location, and the surgical site was sutured with 0–4 Nylon sutures.

All rats received 48% trimethoprim antibiotic at a dosage of 800 mg/kg for 3 days, which was administered intramuscularly or subcutaneously. Meloxicam (1 mg/kg) was also injected intramuscularly once daily for 3 days.

The rats were then randomly assigned to 6 groups (*n* = 6) by block randomization, as follows:

Group 1: The rats did not undergo surgery or laser irradiation.

Group 2: The rats underwent surgery but did not undergo laser irradiation.

Group 3: The rats underwent 808 nm laser (Klas-DX82, Konftec, New Taipe, Taiwan) irradiation with 250 mW power, 0.4 W/cm^2^ power density, 20 s of irradiation time, 8 J/cm^2^ energy density, and 0.5 cm^2^ surface area of the tip after surgery.

Group 4: The rats underwent 660 nm laser (Klas-DX61, Konftec, New Taipe, Taiwan) irradiation with 150 mW power, 0.25 W/cm^2^ power density, 32 s of irradiation time, 8 J/cm^2^ energy density, and 0.5 cm^2^ surface area of the tip after surgery.

Group 5: The rats underwent 808 nm laser irradiation with 250 mW power, 0.4 W/cm^2^ power density, 10 s of irradiation time, 4 J/cm^2^ energy density, and 0.5 cm^2^ surface area of the tip and 660 nm laser irradiation with 150 mW power, 0.25 W/cm^2^ power density, 16 s of irradiation time, 4 J/cm^2^ energy density, and 0.5 cm^2^ surface area of the tip after surgery.

Group 6: The rats underwent 808 nm laser irradiation with 250 mW power, 0.4 W/cm^2^ power density, 20 s of irradiation time, 8 J/cm^2^ energy density, and 0.5 cm^2^ surface area of the tip and 660 nm laser irradiation with 150 mW power, 0.25 W/cm^2^ power density, 32 s of irradiation time, 8 J/cm^2^ energy density, and 0.5 cm^2^ surface area of the tip after surgery (Table 1).

Laser irradiations started the day after surgery for a period of 16 days once every 48 h in continuous mode with a beam diameter of 8 mm.

### 2.3. Histological Assessment

At the end of day 16, the rats were generally anesthetized, the skin of the right side of the face was elevated, and a 5-mm biopsy sample was obtained from the facial nerve injury site. After coding, the specimens were fixed in 10% formalin, embedded in paraffin, sectioned (longitudinal and transverse sections of the nerve), and stained with hematoxylin and eosin by immersion in 1% toluidine blue. The specimens were then inspected under a light microscope (Olympus, Tokyo, Japan) at ×400 magnification. The number of inflammatory cells, the number of Schwann cells, myeline sheath, and blood vessels were all assessed.

### 2.4. Histometric Assessment

The effect of laser on nerve fiber density (fibers/nm^2^) was evaluated. For this purpose, each slide was photographed under a light microscope (BX-41; Olympus, Tokyo, Japan) connected to a camera (Olympus, DP12-z; Tokyo, Japan) such that the entire fascicle was photographed. Next, 5 parts of each fascicle were randomly and systemically selected at ×400 magnification. Of each selected square, one part measuring 350 × 350 μm^2^ was selected, and the mean nerve fiber density was calculated. The micrographs were coded according to the slide coding, saved in a computer, and analyzed by AutoCAD Adobe Photoshop software 2025 v26.0. All specimens were standardized in terms of length, width, and pixels.

### 2.5. Histochemical Analysis

For this purpose, 10-μm sections were made of paraffin blocks of the facial nerve biopsy sample, and mounted on slides. The slides were incubated in 10 mM citrate buffer for 20 min in a microwave, and were then incubated in 0.5% hydrogen peroxide for 15 to 20 min. They were coated with 2% bovine serum albumin for 15 min, and incubated with S100 specific antibody overnight. The concentration of each antibody was determined according to primary tests. The slides were exposed to biotin-conjugated secondary antibody for 30 min, and rinsed with phosphate buffered saline 3 times, if required. The slides were incubated with chromogen for 5 to 10 min, and rinsed with distilled water three times for 5 min.

Background staining with hematoxylin was performed for 10 s. After rinsing with distilled water, they were coated with immunohistochemistry adhesive and stored for imaging.

The mean percentage ratio of the positively stained cells to the total cell count (positive and negative cells) in at least 3 microscopic fields was calculated under a light microscope at ×400 magnification. All assessments were performed by a pathologist blinded to the group allocation of specimens. The following scoring system was used:

Score 0: No staining of the counted cells.
Score 1: Staining of 25% of the counted cells.
Score 2: Staining of 25%–50% of the counted cells.
Score 3: Staining of 50%–75% of the counted cells.
Score 4: Staining of 75%–100% of the counted cells.

### 2.6. Statistical Analysis

Data were analyzed by SPSS version 21 (SPSS Inc., IL, USA). For normally distributed data, general comparisons were made by ANOVA while pairwise comparisons were performed by independent *t*-test. For non-normally distributed data, general comparisons were made by the Kruskal–Wallis test followed by pairwise comparisons with the Wilcoxon signed rank test. *p* < 0.05 was considered statistically significant.

## 3. Results

### 3.1. Histological Results

**Figure 1:**
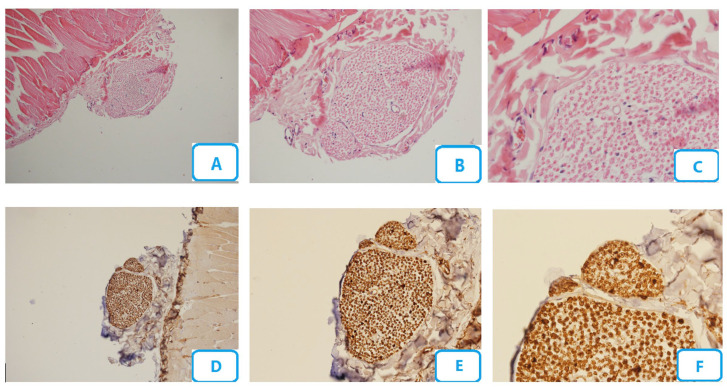
Histological micrographs of the control group; (**A**–**C**) H and E staining with ×10, ×20, and ×40 magnification, respectively; (**D**–**F**) S100 staining with ×10, ×20, and ×40 magnification, respectively.

Figure 1 shows the histological micrographs of the control group. No edema or congestion were seen in this group. There were no nerve fiber degenerative changes, and the number of blood vessels was normal. A small number of Schwann cells were seen, with no degenerative changes. Small numbers of neutrophils, macrophages, and mast cells were also seen, with a high number of myelinated fibers.

**Figure 2:**
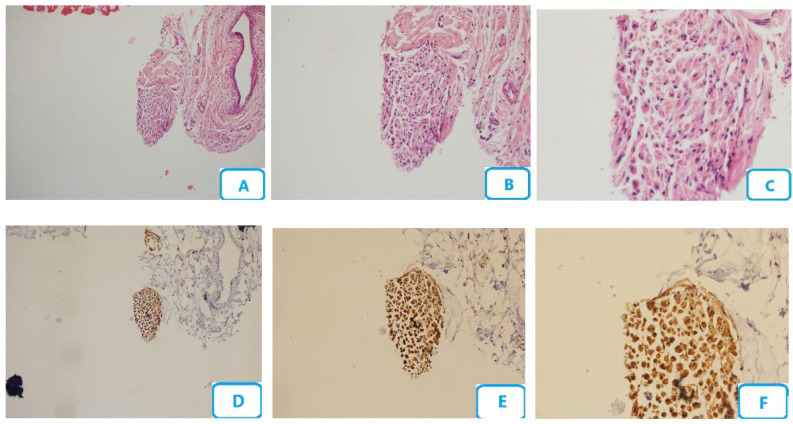
Histological micrographs of the no-laser group; (**A**–**C**) H and E staining with ×10, ×20, and ×40 magnification, respectively; (**D**–**F**) S100 staining with ×10, ×20, and ×40 magnification, respectively.

Figure 2 shows the histological micrographs of the no laser group. Edema, congestion, degenerative changes of the nerve fibers, and number of blood vessels were the highest in this group. A high accumulation of Schwann cells with degenerative changes was also seen. The accumulation of neutrophils, lymphocytes, and macrophages was the highest. No mast cell was seen, and the number of myelinated fibers was the minimum.

**Figure 3:**
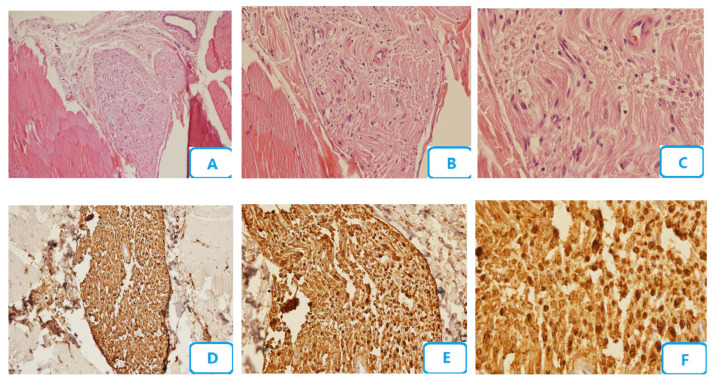
Histological micrographs of 808 nm laser group with 20 s irradiation time and 8 J/cm^2^ energy density; (**A**–**C**) H and E staining with ×10, ×20, and ×40 magnification, respectively; (**D**–**F**) S100 staining with ×10, ×20, and ×40 magnification, respectively.

Figure 3 illustrates the histological micrographs of 808 nm laser group with 20 s irradiation time and 8 J/cm^2^ energy density. Congestion, edema, degenerative changes of the nerve fibers, number of blood vessels, the accumulation of Schwann cells, and the accumulation of neutrophils, lymphocytes, and macrophages were lower than those in the no-laser group. Mast cells were also seen, unlike in the two abovementioned groups. The number of myelinated fibers was higher than that in the no-laser group.

**Figure 4:**
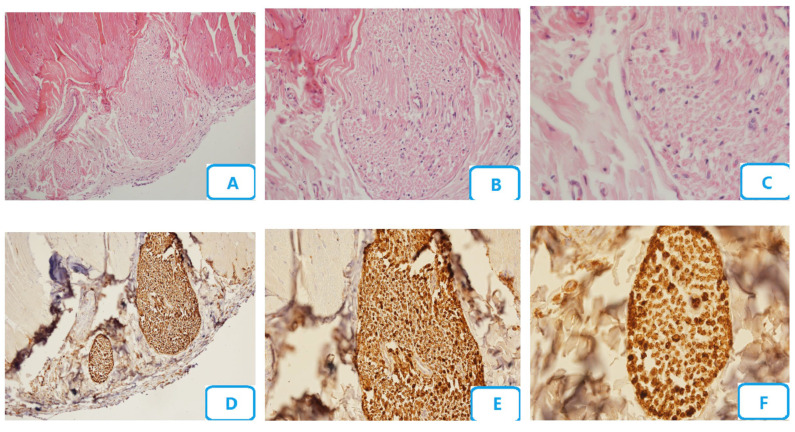
Histological micrographs of the 660 nm laser group with 20 s irradiation time and 8 J/cm^2^ energy density; (**A**–**C**) H and E staining with ×10, ×20, and ×40 magnification, respectively; (**D**–**F**) S100 staining with ×10, ×20, and ×40 magnification, respectively.

Figure 4 illustrates the histological micrographs of the 660 nm laser group with 20 s irradiation time and 8 J/cm^2^ energy density. Edema and congestion in this group were lower than those in the 808 nm laser group, while degenerative changes of the nerve fibers were equal to those in the 808 nm laser group and lower than those in the no-laser group. The number of blood vessels was also lower than that in the no-laser and 808 nm laser groups. The number of Schwann cells and their degenerative changes, and the number of neutrophils, lymphocytes, and macrophages, were also lower than those in the no-laser and 808 nm laser groups. The number of mast cells was equal to that in the 808 nm laser group, while the number of myelinated fibers was higher than that in the no-laser and 808 nm laser groups.

**Figure 5:**
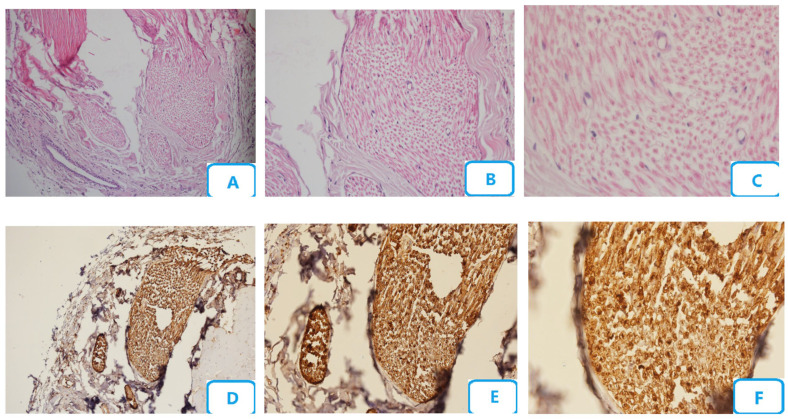
Histological micrographs of 808 nm and 660 nm combined laser group with half the time (10 s) and energy density (4 J/cm^2^); (**A**–**C**) H and E staining with ×10, ×20, and ×40 magnification, respectively; (**D**–**F**) S100 staining with ×10, ×20, and ×40 magnification, respectively.

Figure 5 shows the histological micrographs of the combined laser group with half the time (10 s) and energy density (4 J/cm^2^). Congestion in this group was lower than that in the no-laser, 808 nm, and 660 nm laser groups. Edema was lower than that in the no-laser and 808 nm laser groups, and equal to that in the 660 nm laser group. Degenerative changes of the nerve fibers and Schwann cells, and the accumulation of neutrophils, lymphocytes, and macrophages, were lower than those in the no-laser, 808 nm laser, and 660 nm laser groups. The number of blood vessels was normal. The accumulation of Schwann cells was less than that in the no-laser and 808 nm laser groups, and equal to that in the 660 nm laser group. No mast cells were seen. The number of myelinated fibers was higher than that in the no-laser, 808 nm, and 60 nm laser groups, and lower than that in the control group.

**Figure 6:**
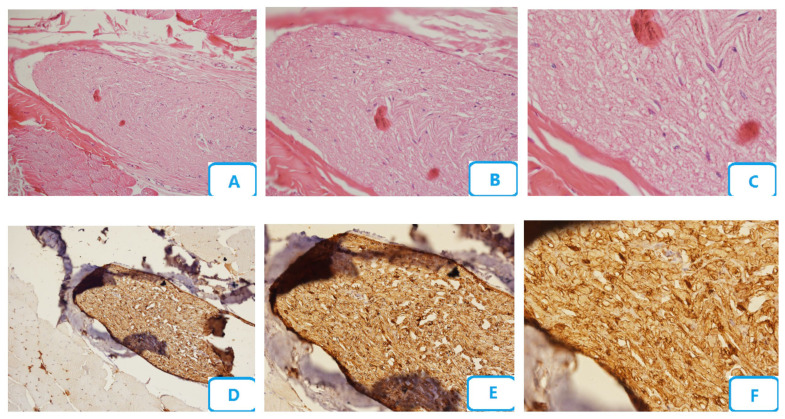
Histological micrographs of 808 nm and 660 nm combined laser group with original time (20 s) and energy density (8 J/cm^2^); (**A**–**C**) H and E staining with ×10, ×20, and ×40 magnification, respectively; (**D**–**F**) S100 staining with ×10, ×20, and ×40 magnification, respectively.

Figure 6 shows the histological micrographs of the combined laser group with original time (20 s) and energy density (8 J/cm^2^). Congestion, degenerative changes of the nerve fibers, the accumulation of Schwann cells, and their degenerative changes were lower than those in the no-laser and the abovementioned three laser groups. Edema was lower than that in the no-laser, 808 nm laser, and 660 nm laser groups, and equal to the combined laser group with half the time and energy density. The number of blood vessels was normal. The accumulation of neutrophils and lymphocytes was lower than that in the no-laser and the above-mentioned laser groups, and the number of macrophages was lower than that in the no-laser, 808 nm laser, and 660 nm laser groups, and equal to that in the combined laser group with half the settings. No mast cells were seen. The number of myelinated fibers was higher than that in all injured groups and lower than that in the control group.

### 3.2. Effect of PBMT on Edema and Congestion

As shown in Table 2, significant differences were found in the scores of edema (*p* < 0.001) and congestion (*p* < 0.001) among the groups. The edema score was the highest in the no-laser and the lowest in the control group. The edema score of the combined laser group with original settings was significantly lower than that in all other groups (*p* < 0.05) except for the control group (*p* > 0.05). The same trend was seen for the congestion score. Combined laser groups showed significantly lower congestion than the no-laser group (*p* < 0.05), and showed no significant difference with the control group (*p* > 0.05).

Effect of low-level lasers on degenerative changes of the nerve fibers and number of blood vessels:

As shown in Table 3, the no-laser group showed the highest, and the control group showed the lowest score for degenerative changes of the nerve fibers (*p* < 0.001). The combined laser group with original settings had no significant difference with the control group (*p* > 0.05), but other groups had a significantly higher score than the control group regarding degenerative changes of the nerve fibers (*p* < 0.05).

Both combined laser groups showed significantly lower number of blood vessels than the no-laser group (*p* < 0.05) but had no significant difference with the control group in this regard (*p* > 0.05).

### 3.3. Effect of PBMT on Accumulation of Schwann Cells and Their Degenerative Changes

As indicated in Table 4, the no-laser group showed the highest, and the control group showed the lowest accumulation of Schwann cells. The 660 nm and combined laser groups had no significant difference with the control group (*p* > 0.05), and showed significantly lower accumulation than other groups (*p* < 0.05).

Only the combined laser group with original settings had no significant difference with the control group regarding degenerative changes (*p* > 0.05), and all other groups had significantly higher degenerative changes than the control group.

Effect of low-level lasers on accumulation of neutrophils, lymphocytes, macrophages, and mast cells (Table 5):

Neutrophils: The control group showed the lowest, and the no-laser group showed the highest accumulation of neutrophils (*p* < 0.05). The control group revealed significantly lower accumulation of neutrophils than other groups (*p* < 0.05). Other groups had no significant difference with each other (*p* > 0.05).

Lymphocytes: The control group had the lowest, and the no-laser group had the highest accumulation of lymphocytes (*p* < 0.05). The control group had significantly lower accumulation of lymphocytes than other groups (*p* < 0.05). Other groups had no significant difference with each other (*p* > 0.05).

Macrophages: The control group had the lowest, and the no-laser group had the highest accumulation of macrophages (*p* < 0.05). The no-laser group showed significantly higher accumulation of macrophages than other groups (*p* < 0.05). The remaining groups had no significant difference with each other (*p* > 0.05).

Mast cells: Mast cells were not seen in the control, no-laser, and combined laser groups, and these groups had no significant difference with each other in this regard (*p* > 0.05). The 808 nm and 660 nm laser groups had a significantly higher percentage of mast cells than other groups (*p* < 0.05).

### 3.4. Effect of PBMT on the Number of Myelinated Fibers (Table 6)

The highest number of myelinated fibers were seen in the control and combined laser groups; the lowest number of myelinated fibers was seen in the no-laser, and 808 and 660 nm laser groups, and this difference was significant (*p* < 0.05).

## 4. Discussion

This study assessed the efficacy of 808 nm and 630 nm low-level lasers alone and in combination for treatment of paresthesia in rats. The results showed significantly lower edema and congestion in combined laser group with original parameters, highlighting the anti-inflammatory effect of this combination. This group also had no significant difference with the control group regarding degenerative changes of the nerve fibers, while other groups had significantly higher scores than the control group. The 660 nm and combined laser groups showed a significantly lower accumulation of inflammatory cells. The no-laser group showed the highest accumulation of neutrophils, lymphocytes, and macrophages, and other groups had no significant difference with each other in this respect. Also, the 808 and 660 nm laser groups showed a significantly higher accumulation of mast cells. The results revealed the positive efficacy of 808 nm and 660 nm lasers in the resolution of inflammation and the reduction of degenerative changes of the Schwann cells and nerve fibers.

Evidence shows that PBMT can decrease the release of inflammatory cytokines such as prostaglandin E2, cyclooxygenase 2, and interleukin-1B. It can also decrease the neutrophil infiltration and edema after soft tissue injury, and can control inflammation as such. Laser therapy decreases the activity of sensory nerves, and the local inflammatory response, preventing vascular dilation and edema in cases of inflammation and acute injury [18,19]. In line with the present results, Giuliani et al. [20] used PBMT in rats with nerve injury and reported that it effectively decreased edema and hyperalgesia in acute and chronic inflammations.

To the best of the authors’ knowledge, no previous study has addressed the effect of combined use of different low-level laser wavelengths on edema and congestion. Different laser wavelengths have different penetration depths into the tissue; the 808 nm laser is within the near infra-red range, and can penetrate deeper into the tissue while 660 nm wavelength is in the range of red light, and is more suitable for more superficial tissues. The combination of these two wavelengths affects both superficial and deep tissues, resulting in more efficient resolution of inflammation and edema. Also, the combined use of two wavelengths may exert synergistic effects, and lead to the further reduction of edema and congestion, because different biological mechanisms are induced by different laser wavelengths [21].

The number of blood vessels in combined laser groups was significantly lower than that in the no-laser group. Barez et al. [22] indicated that PBMT accelerated revascularization and enhanced angiogenesis at the site of sciatic nerve injury in rats, which was in line with the present findings. Also, Ma et al. [23] reported that the 810 nm laser significantly increased the number of arterioles and capillaries in ischemic flaps compared to the control group. A lack of a significant difference between combined laser groups and the control group in this regard in the current study may indicate returning to a normal state and the resolution of inflammation.

In the current study, the score of degenerative changes of the nerve fibers in the combined laser group with original settings was comparable to that in the control group, also, the highest number of myelinated fibers was seen in the combined laser groups. Consistent with the present results, Sene et al. [24] reported significantly higher density of nerve fibers in rats with compressive injury to the fibular nerve subjected to irradiation of the 830 nm laser with 10 J/cm^2^ energy density. Also, Wang et al. [6] showed that PBMT with the 808 nm laser with 3 and 8 J/cm^2^ energy densities significantly improved the nerve function and increased the myelin sheath thickness of injured sciatic nerve in rats. Gigo-Benato et al. [25] demonstrated that the 660 nm laser with 10 and 60 J/cm^2^ energy densities effectively increased the cross-sectional area of nerve fibers, axons, and myelin sheath. Ziago et al. [26] reported improvement in morphological, quantitative, and morphometric parameters in rats with sciatic nerve injury following irradiation of the 780 nm laser especially with 10 J/cm^2^ energy density. The decreased destruction of nerve fibers in the combined laser groups in the present study can be due to the combined use of two wavelengths with lower energy density, which may activate higher number of compensatory and protective mechanisms, as well as the biostimulatory effects of lower doses explained by the Arndt-Schulz rule [27,28].

The combined laser group with original settings was comparable to the control group regarding degenerative changes of Schwann cells. Accumulation of Schwann cells in the 660 nm and combined laser groups was also comparable to that in the control group. This result was in agreement with the findings of Yazdani et al. [29], Andero et al. [30] (who used 660 nm and 780 nm lasers), and Câmara et al. [31].

The present results found no significant difference among the laser groups in the number of inflammatory cells. Evidence shows that PBMT decreases the inflammation and controls the accumulation of neutrophils and lymphocytes at the site of injury [32,33]. The higher accumulation of macrophages in the no-laser group is probably due to normal inflammatory response to injury. PBMT can modulate this response [34], as seen in the laser groups. A higher number of mast cells were seen in the 808 and 660 nm laser groups. PBMT reduces the synthesis of pro-inflammatory cytokines, including tumor necrosis factor-alpha (TNF-α) and interleukin-6 (IL-6), while simultaneously promoting the production of anti-inflammatory cytokines such as IL-10. This modulation fosters an ideal environment for nerve repair by mitigating damage caused by inflammation [35]. PBMT stimulates the release of nerve growth factors (NGFs), such as brain-derived neurotrophic factor (BDNF), which promote axonal sprouting and repair. It also enhances the proliferation of Schwann cells, critical for myelination of regenerated nerve fibers [36].

No previous study is available in this regard to compare our results with, and future studies are required in this regard. It is essential to highlight that the findings of this animal study should not be directly applied to clinical environments. Also, it is suggested that the long-term effects of PBM treatment in animal studies are evaluated by increasing relevant molecular biological tests.

## 5. Conclusions

The results showed the positive efficacy of PBMT by 808 nm and 660 nm lasers in the resolution of inflammation and the reduction of degenerative changes of the Schwann cells and nerve fibers.

## Figures and Tables

**Table 1:** Laser parameters used.

Wavelength (nm)	Power (mW)	Tip Diameter (mm)	Power Density (W/cm^2^)	Time of Irradiation (s)	Energy Density (J/cm^2^)
808	250	8	0.4	20	8
660	150	8	0.25	32	8
808 + 660	250 + 150	8	0.4 + 0.25	10 + 16	4 + 4
808 + 660	250 + 150	8	0.4 + 0.25	20 + 32	8 + 8

**Table 2:** Scores for the edema and congestion in the study groups. In the comparison among groups, they have been designated by letters. Groups that were assigned different letters exhibited a statistically significant difference (*p* < 0.05).

		Congestion Scores			Edema Scores		
		Mean	Standard Deviation	*p* Value	Mean	Standard Deviation	*p* Value
Groups	Control	0.00 ^a^	0.00	<0.001	0.00 ^a^	0.00	<0.001
	No laser therapy	2.40 ^b^	0.89		2.67 ^b^	0.52	
	808 nm (8 J/cm^2^)	1.40 ^b^	0.55		2.17 ^bc^	0.41	
	660 nm (8 J/cm^2^)	2.00 ^b^	0.71		1.17 ^c^	0.41	
	808 + 660 nm (4 J/cm^2^ + 4 J/cm^2^)	0.80 ^a^	0.45		1.17 ^c^	0.41	
	808 + 660 nm (8 J/cm^2^ + 8 J/cm^2^)	0.40 ^a^	0.55		0.33 ^a^	0.52	

**Table 3:** Scores for the degenerative changes of the nerve fibers and number of blood vessels in the study groups. In the comparison among groups, they have been designated by letters. Groups that were assigned different letters exhibited a statistically significant difference (*p* < 0.05).

		Degenerative Changes of the Nerve Fibers Scores			Number of Blood Vessels Scores		
		Mean	Standard Deviation	*p* Value	Mean	Standard Deviation	*p* Value
Groups	Control	0.00 ^a^	0.00	<0.001	0.00 ^a^	0.00	<0.001
	No laser therapy	2.50 ^b^	0.55		2.50 ^b^	0.55	
	808 nm (8 J/cm^2^)	1.83 ^b^	0.41		1.50 ^b^	0.55	
	660 nm (8 J/cm^2^)	1.83 ^b^	0.41		0.50 ^b^	0.55	
	808 + 660 nm (4 J/cm^2^ + 4 J/cm^2^)	1.33 ^b^	0.52		0.00 ^a^	0.00	
	808 + 660 nm (8 J/cm^2^ + 8 J/cm^2^)	0.17 ^a^	0.41		0.00 ^a^	0.00	

**Table 4:** Scores for the accumulation of Schwann cells and their degenerative changes in the study groups. In the comparison among groups, they have been designated by letters. Groups that were assigned different letters exhibited a statistically significant difference (*p* < 0.05).

		Schwann Cells Degenerative Changes Score			Accumulation of Schwann Cells Score		
		Mean	Standard Deviation	*p* Value	Mean	Standard Deviation	*p* Value
Groups	Control	0.00 ^a^	0.00	<0.001	0.00 ^a^	0.00	<0.001
	No laser therapy	2.50 ^b^	0.55		2.17 ^b^	1.17	
	808 nm (8 J/cm^2^)	1.83 ^b^	0.41		1.33 ^b^	1.03	
	660 nm (8 J/cm^2^)	1.80 ^b^	0.41		0.33 ^a^	0.52	
	808 + 660 nm (4 J/cm^2^ + 4 J/cm^2^)	1.33 ^b^	0.52		0.33 ^a^	0.52	
	808 + 660 nm (8 J/cm^2^ + 8 J/cm^2^)	0.17 ^a^	0.41		0.17 ^a^	0.41	

**Table 5:** Scores for the accumulation of neutrophils, lymphocytes, macrophages, and mast cells in the study groups. In the comparison among groups, they have been designated by letters. Groups that were assigned different letters exhibited a statistically significant difference (*p* < 0.05).

		Neutrophil			Lymphocyte			Macrophage			Mast Cell		
		Mean	Standard Deviation	*p* Value	Mean	Standard Deviation	*p* Value	Mean	Standard Deviation	*p* Value	Mean	Standard Deviation	*p* Value
Groups	Control	0.00 ^a^	0.00	<0.001	0.00 ^a^	0.00	<0.001	0.00 ^a^	0.00	<0.001	0.00 ^a^	0.00 ^a^	<0.001
	No laser therapy	1.67 ^b^	1.21		3.50 ^b^	5.47		2.17 ^b^	0.75		0.00 ^a^	0.00 ^a^	
	808 nm (8 J/cm^2^)	1.00 ^b^	1.10		1.17 ^b^	1.17		0.50 ^a^	0.84		0.17 ^b^	0.41 ^b^	
	660 nm (8 J/cm^2^)	0.17 ^b^	0.41		0.98 ^b^	1.1		0.50 ^a^	0.84		0.17 ^b^	0.41 ^b^	
	808 + 660 nm (4 J/cm^2^ + 4 J/cm^2^)	0.50 ^b^	0.84		0.87 ^b^	0.41		0.17 ^a^	0.41		0.00 ^a^	0.00 ^a^	
	808 + 660 nm (8 J/cm^2^ + 8 J/cm^2^)	0.33 ^b^	0.52		0.33 ^b^	0.52		0.17 ^a^	0.41		0.00 ^a^	0.00 ^a^	

**Table 6:** Number of myelinated fibers in the study groups. In the comparison among groups, they have been designated by letters. Groups that were assigned different letters exhibited a statistically significant difference (*p* < 0.05).

		Number of Myelinated Fibers		
		Mean	Standard Deviation	*p* Value
Groups	Control	1.00 ^a^	0.00	<0.001
	No laser therapy	0.17 ^b^	0.41	
	808 nm (8 J/cm^2^)	0.50 ^a^	0.55	
	660 nm (8 J/cm^2^)	0.50 ^a^	0.55	
	808 + 660 nm (4 J/cm^2^ + 4 J/cm^2^)	0.67 ^a^	0.52	
	808 + 660 nm (8 J/cm^2^ + 8 J/cm^2^)	0.83 ^a^	0.41	

## Data Availability

The datasets used and/or analyzed during the current study are available from the corresponding author on reasonable request.
